# Non-communicable disease burden among inpatients at a rural district hospital in Malawi

**DOI:** 10.1186/s41256-023-00289-z

**Published:** 2023-02-22

**Authors:** Peter Olds, Chiyembekezo Kachimanga, George Talama, Bright Mailosi, Enoch Ndarama, Jodie Totten, Nicholas Musinguzi, Dickson Hangiwa, Gene Bukhman, Emily B. Wroe

**Affiliations:** 1 Massachusetts General Hospital, Boston, MA USA; 2 Harvard Medical School, Boston, MA USA; 3 Partners in Health, Neno, Malawi; 4 Ministry of Health and Population, Lilongwe, Malawi; 5 Department of Emergency Medicine, University of Washington, Seattle, WA USA; 6 Global Health Collaborative, Mbarara University of Science and Technology, Mbarara, Uganda; 7 Center for Integration Science, Division of Global Health Equity, Brigham and Women’s Hospital, Boston, MA USA; 8 Program in Global NCDs and Social Change, Deparment of Global Health and Social Medicine, Harvard Medical School, Boston, MA USA

**Keywords:** Malawi, Non-communicable disease, Burden of disease, Inpatient, Rural, Hospital

## Abstract

**Background:**

The burden of non-communicable diseases (NCDs) is high in Malawi. However, resources and training for NCD care remain scarce, especially in rural hospitals. Current care for NCDs in the developing world focuses on the WHO’s traditional 4 × 4 set. However, we do not know the full burden of NCDs outside of that scope, like neurological disease, psychiatric illness, sickle cell disease, and trauma. The goal of this study was to understand the burden of NCDs among inpatients in a rural district hospital in Malawi. We broadened our definition of NCDs beyond the traditional 4 × 4 set of NCDs, and included neurological disease, psychiatric illness, sickle cell disease, and trauma.

**Methods:**

We conducted a retrospective chart review of all inpatients who were admitted to the Neno District Hospital between January 2017 and October 2018. We broke patients down by age, date of admission, type, and number of NCD diagnoses, and HIV status, and constructed multivariate regression models for length of stay and in-hospital mortality.

**Results:**

Of 2239 total visits, 27.5% were patients with NCDs. Patients with NCDs were older (37.6 vs 19.7 years, *p* < 0.001) and made up 40.2% of total hospital time. We also found two distinct populations of NCD patients. The first were patients 40 years and older with primary diagnoses of hypertension, heart failure, cancer, and stroke. The second were patients under 40 years old with primary diagnoses of mental health conditions, burns, epilepsy, and asthma. We also found significant trauma burden, accounting for 40% of all NCD visits. In multivariate analysis, carrying a medical NCD diagnosis was associated with longer length of stay (coefficient 5.2, *p* < 0.001) and a higher risk of in-hospital mortality (OR 1.9, *p* = 0.03). Burn patients also had significantly longer length of stay (coefficient 11.6, *p* < 0.001).

**Conclusions:**

There is a significant burden of NCDs in a rural hospital in Malawi, including those outside of the traditional 4 × 4 set. We also found high rates of NCDs in the younger population (under 40 years of age). Hospitals must be equipped with adequate resources and training to meet this burden of disease.

**Supplementary Information:**

The online version contains supplementary material available at 10.1186/s41256-023-00289-z.

## Introduction

Non-communicable diseases (NCDs) are a growing health burden in Sub-Saharan Africa (SSA) [1] with deaths from NCDs projected to outpace infectious causes by 2030 [2]. This has led to a dual burden of disease in SSA, where countries face an ongoing epidemic of communicable diseases like human immunodeficiency virus (HIV), tuberculosis (TB), and malaria, as well as a dramatic rise in the burden of NCDs [3]. Despite the burden of disability-adjusted life year (DALY) due to NCDs estimated at 37% in low-income countries, only an estimated 1.5% of development assistance in 2015 was allocated to combat NCDs [4].

The burden of NCDs is especially significant in Malawi, where there is already a high incidence of NCDs and their risk factors. In Malawi, rates of heart disease and diabetes are 9% and 6% respectively [5] and the prevalence hypertension, obesity, and smoking are estimated at 16.7%, 18.5%, and 21.7% respectively [6]. And while NCDs in the traditional set (cardiovascular disease, diabetes, chronic respiratory diseases, and cancers) are rising [7], there is a significant burden of NCDs outside the traditional set, including relatively high impact of neurologic disease, psychiatric illness, trauma, and other complex chronic conditions such as type 1 diabetes or sickle cell anemia [8–12]. In Malawi, disease burden has also significantly outpaced funding, with only 0.1% of programmatic funding in 2015–2016 designated towards NCDs [13].

In working to address the NCD epidemic, the Ministry of Health organized the Malawi NCDI Poverty Commission [14], who, with the support of the Lancet NCDI (non-communicable diseases and injuries) Poverty Commission [15], are focused on broadening the approach to NCDs beyond the traditional set as outlined by the World Health Organization (WHO) Package of Essential Noncommunicable Disease Interventions (WHO PEN) [16]. This approach, termed PEN-Plus, focuses not only on the traditional set of NCDs and their behavioral risk factors, but also includes the provision of care at district hospitals for severe chronic NCDs such as type 1 diabetes, rheumatic heart disease (RHD), sickle cell disease, neurologic disease, among others. Additionally, PEN-Plus aims to provide resources to treat patients with advanced disease not adequately treated by WHO PEN [17].

The WHO’s goal for Universal Health Coverage (UHC) by 2030 [18] pushes for universal access to essential healthcare services while also providing adequate financial risk protection to patients. Providing access to care for NCDs, especially for conditions encompassed by PEN-Plus, is a critical part of reaching UHC [15] and remains a major barrier to reaching UHC [19]. Understanding the true burden of NCDs is a critical first step towards improving patient care, increasing access, strengthening patient financial risk protection, and achieving UHC.

Acute care hospitals see a significant portion of the rising NCD disease burden in SSA, with increasing numbers of patients arriving with NCDs, especially in late stages. In Nigeria, Sudan, and Tanzania, NCDs accounted for 81% of inpatient admissions in patients over 60 years old [20]. At an urban referral hospital in Blantyre, Malawi, NCDs were the most common diagnoses and the leading cause of death in patients over 55 years old [21]. Importantly, hospitals and health systems in SSA are currently ill-equipped to handle this surge in NCD patients [2, 22], highlighting the importance of better understanding the true burden of disease so as to better address the epidemic.

While prior studies have focused largely on urban populations, data shows that the prevalence of many NCDs is similarly high in rural areas [7] and on the rise [23]. Additionally, these studies likely underestimate the true burden of NCDs facing rural health systems, as they focus on the traditional set of hypertension, diabetes, asthma, and cancers, without a broader look at trauma and injuries, severe mental health, and neurological conditions that are more likely to affect young people. Additionally, rural hospitals face a more significant lack of infrastructure, training, and resources necessary to properly diagnose and treat NCDs than their urban counterparts. Improving care at rural hospitals is especially important in Malawi, where 84% of the population lives in rural areas [24]. In this paper, we describe the burden of NCDs and injuries among all inpatients in a rural hospital in the Neno district in southwestern Malawi.

## Methods

### Setting

This study was conducted at Neno District Hospital in Neno, Malawi. The hospital is a 172-bed hospital with adult, pediatrics, obstetric, and tuberculosis wards. The hospital serves the entire Neno district, which is located in southwest Malawi with a population of approximately 140,000 in 2018 [24]. The district has one community hospital and 12 health centers that refer patients to Neno District Hospital. Neno district is remote and impoverished, with a majority of the population relying on subsistence farming, and only 4.5% of the population with access to electricity [25]. Since 2007, Partners In Health (PIH) has been working with the MOH to strengthen both primary and secondary healthcare services [26–30].

In partnership with PIH, Neno district has longitudinal integrated chronic care clinics (IC3) caring for HIV and chronic NCDs, focused on services laid out by WHO PEN [16]. These clinics were initially based at the two district hospitals, but now care for more than 5,000 patients with chronic NCDs at 14 sites across the district [31]. Retention at one year after enrollment for patients with NCDs is more than 70% [25]. Since 2018, Neno also has two hospital-based clinics for patients with severe or complex NCDs requiring a higher level of longitudinal outpatient care. These clinics, defined by PEN-Plus, have allowed a broad set of NCDs to be treated effectively in low resource settings [32–35]. With the expertise from these NCD clinics, there is ongoing staff training and increasing resources for improving both inpatient and outpatient NCD care throughout the district, including staff training and mentorship, case finding and linkage to care, provision of socioeconomic support, and monitoring and evaluation.

### Research design

The objective of this study was to assess the burden of NCDs among patients admitted to the Neno District Hospital in rural Malawi. We performed a retrospective review of inpatients admitted to the Neno District Hospital for a 22-month period between Jan 2 2017 and October 26 2018. All patients were included who had been admitted to the male, female, pediatric, and tuberculosis (TB) wards of the hospital. We constructed a multivariate linear regression model for factors associated with patient hospital length of stay and a multivariate logistic regression model for factors associated with in-hospital mortality.

### Data collection

Data were extracted from paper charts and included basic demographics, date of admission, admission vital signs, length of stay, HIV status, TB diagnosis, malaria diagnosis, enrollment in the IC3 clinic, presence of chronic medical NCDs, injuries, trauma, need for surgery, hospital transfer, and mortality. Diagnosis of NCD was based on clinical diagnosis made by clinicians of Neno District Hospital and documented in the medical chart. Systemic Inflammatory Response Syndrome was defined as having two or more of the following variables: temperature over 38 or under 36 °C, heart rate greater than 90 beats/minute, respiratory rate greater than 20 breaths/min. Hypotension was defined as a systolic blood pressure under 90 mmHg, and hypoxia was defined as an oxygen saturation less than 90%. We conducted HIV testing on all inpatients who didn’t have a confirmed HIV status, or those who wanted retesting. Rapid HIV tests supplied by the Malawian Ministry of Health were used for testing according to national guidelines [36]. Gene Xpert (Cepheid, Sunnyvale, CA) was used for assessing HIV viral load. Patients did not have unique identifiers, and we identified readmissions by matching name, age, and home village. Data was stored on encrypted devices and all documents were password protected.

### Data analysis

Participant characteristics were summarized descriptively. Comparisons between patients carrying at least one NCD diagnosis and those without an NCD diagnosis were made with Wilcoxson rank sum and Pearson’s chi-square tests for continuous and categorical variables respectively. Medical NCDs were defined as hypertension, diabetes mellitus, epilepsy, asthma, chronic obstructive pulmonary disease (COPD), congestive heart failure (CHF), rheumatic heart disease (RHD), active cancer, liver disease, sickle cell disease, stroke, chronic kidney disease (CKD), and mental health. Patients with NCDs were further broken down by age into older (≥ 40 years of age) and younger (< 40 years of age) and the younger population was also broken down into young adult (15–39 years of age) and pediatric populations (< 15 years of age). NCD diagnoses by age were summarized descriptively with comparisons between groups calculated by Pearson’s chi-square test. The rainy season was defined as occurring between Dec 1 and March 31 of each year.

All tests were two-sided and a p-value less than 0.05 was considered statistically significant. All variables were initially assessed for significance using univariate analysis comparing length of stay and mortality. A multivariate linear regression was fitted separately comparing length of stay, and a multivariate logistic regression was fitted separately comparing in-hospital mortality. Variables were excluded if they showed significant co-linearity (variance inflation factors over 10). We used stepwise, backward selection for our logistic regression model, using a p-value of over 0.2 as a cut-off to remove variables. Potential interaction between significant variables was explored.

To represent the data visually, we created box plots to evaluate the age distributions for patients with or without an NCD diagnosis as well as age distributions for total number of unique diagnoses a patient carried.

All data were analyzed using Stata Statistical Software (Release 16. College Station, TX: StataCorp LLC). IRB approval for the study was obtained through the Malawian National Health Sciences Research Committee (#1216) and Partners Healthcare IRB in Boston, Massachusetts (Protocol #2014P001460).

## Results

Overall, 2,239 inpatient visits were recorded, of which 27.5% were patients with NCDs. (Table 1) The mean age for all visits was 24.6 years (standard deviation [SD] 22.7), with NCD patients having a mean age of 37.6 (SD 25.2) compared to 19.7 (SD 19.6) for non-NCD patients (*p* < 0.001), shown visually in Additional file 1: Fig. S1. Carrying more than one unique NCD diagnosis was more common in older patients (Additional file 1: Fig. S2). Just over half of the total hospitalizations were female patients (54.1%), while NCD hospitalizations had a slight male predominance (42.8% female).

**Table 1:** Baseline characteristics

Variable	Overall	NCD	Non-NCD	*p* value
Total visits (N, %)	2239 (100)	615 (27.5)	1624 (72.5)	
Age, mean, years (SD)	24.6 (22.7)	37.6 (25.2)	19.7 (19.6)	< 0.001
Female sex (N, %)	1211 (54.1)	263 (42.8)	948 (58.4)	< 0.001
HIV positivity (N, %)	269 (12.0)	67 (10.9)	202 (12.4)	0.32
TB positivity (N, %)	86 (3.8)	23 (3.7)	63 (3.9)	0.88
Length of stay, mean, days (SD)	5.4 (9.9)	7.9 (13.6)	4.4 (7.9)	< 0.001
Percent total hospital time	100	40.2	59.8	0.01
Readmissions (N, %)	103 (4.6)	41 (6.7)	62 (3.8)	0.004
Transfers to higher levels of care (N, %)	86 (3.8)	38 (6.2)	48 (3.0)	< 0.001
Mortality (N, %)	89 (4.0)	39 (6.3)	50 (3.1)	< 0.001
Total tested for HIV (N, %)	1100 (49.1)	295 (48.0)	805 (49.6)	0.50
Positive HIV tests (N, %)	14 (1.3)	0 (0.0)	14 (1.7)	0.02
Surgery (N, %)	252 (11.3)	51 (8.3)	201 (12.4)	0.01

Descriptive statistics of the patient cohort

*NCD* Non-communicable disease, *SD* Standard deviation, *HIV* Human immunodeficiency virus, *TB* Tuberculosis

The overall mean length of stay was 5.4 days (SD 9.9), but NCD clients had longer stay than those without NCDs, 7.9 (SD 13.6) vs 4.4 (SD 7.9) days, respectively (*p* < 0.001). Importantly, NCD patients accounted for 40.2% of total hospital time (*p* = 0.01). NCD patients also had higher readmission rates than non-NCD patients, with rates of 6.7% and 3.8%, respectively (*p* = 0.004). Transfers to higher levels of care were infrequent (4.6% of admissions), though twice as common in patients with an NCD diagnosis (6.2% vs 3.0%, *p* < 0.001).

A significant proportion of our patient population was HIV positive (12.0%), and among NCD hospitalizations, 10.9% had HIV as a co-morbidity. Roughly half of our inpatient population (49.1%) was tested for HIV, and of those, we had a 1.3% positivity rate. This is in line with average positive testing in Malawi, though is lower than our group has found in previous years [37].

**Fig. 1:**
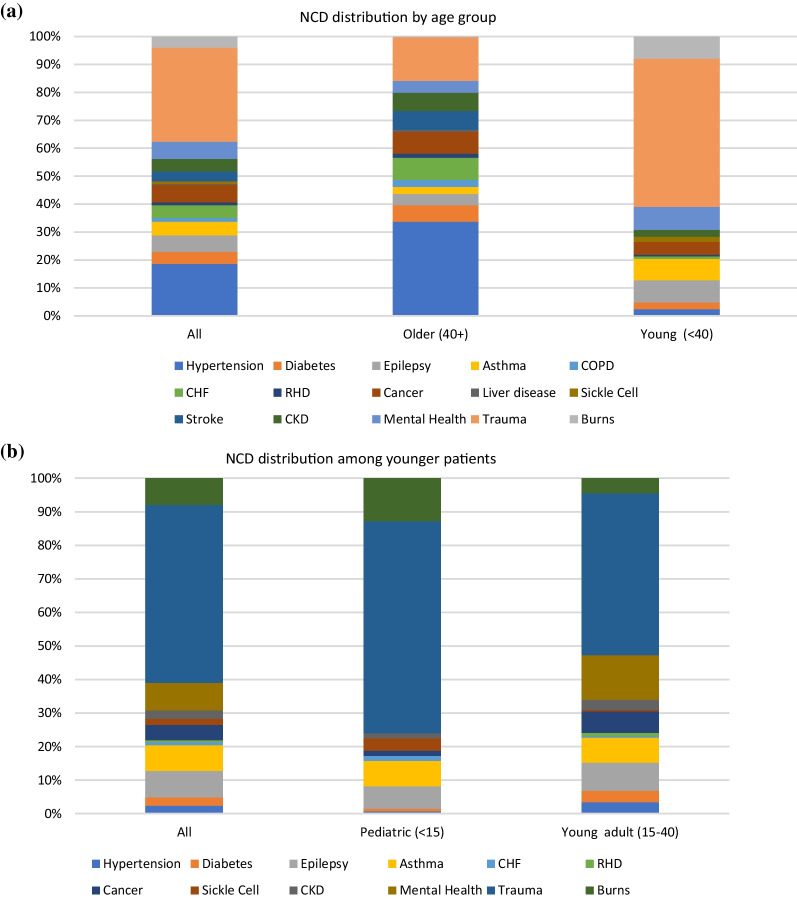
**a** Non-communicable disease distribution by age group. Percent distribution of each NCD among total NCD diagnoses, broken down for all patients, patients 40 years and older, and patients under 40 years of age. *NCD* Non-communicable disease, *COPD* Chronic obstructive pulmonary disease, *CHF* Congestive heart failure, *RHD* Rheumatic heart disease, *CKD* Chronic kidney disease. **b** Non-communicable disease distribution among patients under 40 years of age. Percent distribution of each NCD among total NCD diagnoses, broken down for all patients under 40 years of age, patients under between 15 and 40 years of age, and patients under 15 years of age. *NCD* Non-communicable disease, *CHF* Congestive heart failure, *RHD* Rheumatic heart disease, *CKD* Chronic kidney disease

The type of NCDs in patients over 40 years of age versus those under 40 years of age showed substantial differences (Table 2). The most common NCDs among hospitalized patients over 40 years were hypertension, heart failure, cancer, and stroke. Among patients with NCDs under age 40 the most common NCDs were mental health conditions, burns, epilepsy, and asthma. Trauma was a top contributor across all ages. The differences between these populations are highlighted visually in Fig. 1a.

**Table 2:** Analysis of NCD patients by age group

Variable	All	Older (40 +)	Young (< 40)	*p* value
Total patients (N, %)	574	262	312	
Multiple NCD diagnoses (N, %)	95 (16.6)	80 (30.5)	15 (4.8)	< 0.001
Hypertension (N, %)	127 (22.1)	119 (45.4)	8 (2.6)	< 0.001
Diabetes (N, %)	29 (5.1)	21 (8.0)	8 (2.6)	0.003
Epilepsy (N, %)	40 (7.0)	14 (5.3)	26 (8.3)	0.16
Asthma (N, %)	34 (5.9)	9 (3.4)	25 (8.0)	0.02
COPD (N, %)	9 (1.6)	9 (3.4)	0 (0.0)	0.001
CHF (N, %)	31 (5.4)	28 (10.7)	3 (1.0)	< 0.001
RHD (N, %)	7 (1.2)	5 (1.9)	2 (0.6)	0.17
Cancer (N, %)	43 (7.5)	28 (10.7)	15 (4.8)	0.01
Liver disease (N, %)	2 (0.3)	2 (0.8)	0 (0.0)	0.12
Sickle cell disease (N, %)	6 (1.0)	0 (0.0)	6 (1.9)	0.02
Stroke (N, %)	24 (4.2)	24 (9.2)	0 (0.0)	< 0.001
CKD (N, %)	31 (5.4)	23 (8.8)	8 (2.6)	0.001
Mental health disorder (N, %)	42 (7.3)	15 (5.7)	27 (8.7)	0.18
Trauma (N, %)	229 (39.9)	55 (21.0)	174 (55.8)	< 0.001
Burns (N, %)	27 (4.7)	1 (0.4)	26 (8.3)	< 0.001

*NCD* Non-communicable disease, *COPD* Chronic obstructive pulmonary disease, *CHF* Congestive heart failure, *RHD* Rheumatic heart disease, *CKD* Chronic kidney disease

Among patients with NCDs under age 40, there are additional differences in the pediatric population (< 15 years) as compared to young adults (15–40 years) (Table 3). While trauma was the top contributor in both groups, the next top contributors in children under 15 years were burns, asthma, and epilepsy, while the next contributor for young adults 15–39 years was mental health, and cancer joined epilepsy and asthma as a top condition in this group. The differences between these populations are highlighted visually in Fig. 1b.

**Table 3:** Distribution of NCDs among the younger population

Variable	All	Pediatric (< 15)	Young adult (15–39)	*p* value
All NCD patients	312	129	183	
Multiple NCD diagnoses (N, %)	15 (4.8)	3 (2.3)	12 (6.6)	0.09
Hypertension (N, %)	8 (2.6)	1 (0.8)	7 (3.8)	0.09
Diabetes (N, %)	8 (2.6)	1 (0.8)	7 (3.8)	0.09
Epilepsy (N, %)	26 (8.3)	9 (7.0)	17 (9.3)	0.47
Asthma (N, %)	25 (8.0)	10 (7.8)	15 (8.2)	0.89
COPD (N, %)	0 (0.0)	0 (0.0)	0 (0.0)	n/a
CHF (N, %)	3 (1.0)	2 (1.6)	1 (0.5)	0.37
RHD (N, %)	2 (0.6)	0 (0)	2 (1.1)	0.23
Cancer (N, %)	15 (4.8)	2 (1.6)	13 (7.1)	0.02
Liver disease (N, %)	0 (0.0)	0 (0.0)	0 (0.0)	n/a
Sickle cell disease (N, %)	6 (1.9)	5 (3.9)	1 (0.5)	0.04
Stroke (N, %)	0 (0.0)	0 (0.0)	0 (0.0)	n/a
CKD (N, %)	8 (2.6)	2 (1.6)	6 (3.3)	0.34
Mental health disorder (N, %)	27 (8.7)	0 (0.0)	27 (14.8)	< 0.001
Trauma (N, %)	174 (55.8)	84 (65.1)	98 (53.6)	0.01
Burns (N, %)	26 (8.3)	17 (13.2)	9 (4.9)	0.01

*NCD* Non-communicable disease, *COPD* Chronic obstructive pulmonary disease, *CHF* Congestive heart failure, *RHD* Rheumatic heart disease, *CKD* Chronic kidney disease

Factors that were associated with length of stay on univariate analysis included age (*p* < 0.001), IC3 enrollment (*p* < 0.001), HIV diagnosis (*p* < 0.001), TB diagnosis (*p* < 0.001), malaria diagnosis (*p* < 0.001), medical NCD diagnosis (*p* < 0.001), number of medical NCD diagnoses (*p* < 0.001), and a burns diagnosis (*p* < 0.001) (Table 4). On multivariate analysis, factors that were associated with longer hospital stay included age (coefficient 0.04, *p* < 0.001), TB diagnosis (coefficient 10.1, *p* < 0.001), medical NCD diagnosis (coefficient 5.2, *p* < 0.001), and burns diagnosis (coefficient 11.6, *p* < 0.001). Malaria diagnosis (coefficient − 1.7, *p* = 0.001) and number of medical NCD diagnoses (coefficient − 2.0, *p* = 0.03) were associated with a shorter length of stay.

**Table 4:** Criteria associated with patient length of stay

Variable	Univariate		Multivariate	
	Coefficient (95% CI)	*p* value	Coefficient (95% CI)	*p* value
Age, years (per year)	0.1 (0.1, 0.1)	< 0.001	0.04 (0.02, 0.1)	< 0.001
Female sex	− 0.7 (− 1.5, 0.1)	0.10	− 0.7 (− 1.4, 0.1)	0.07
IC3-enrolled	4.5 (3.3, 5.7)	< 0.001		
Rainy season admission	− 0.2 (− 1.0, 0.6)	0.66		
SIRS on admission	− 0.6 (− 1.7, 0.4)	0.23		
Hypoxia (oxygen saturation < 90%) on admission	1.7 (− 0.6, 4.0)	0.14		
Hypotension (SBP < 90 mmHg) on admission	0.6 (− 1.8, 3.1)	0.63		
HIV diagnosis	3.6 (2.4, 4.9)	< 0.001		
TB diagnosis	11.4 (9.2, 13.5)	< 0.001	10.1 (8.2, 12.0)	< 0.001
Malaria diagnosis	− 3.5 (− 4.6, − 2.6)	< 0.001	− 1.7 (− 2.6, − 0.7)	0.001
Surgery during hospiatlization	0.5 (− 0.8, 1.9)	0.44		
Medical NCD diagnosis	4.6 (3.5, 5.7)	< 0.001	5.2 (2.8, 7.6)	< 0.001
Number of medical NCD diagnoses (per unit)	2.8 (2.0, 3.6)	< 0.001	− 2.0 (− 3.7, − 0.2)	0.03
Burns diagnosis	11.2 (7.4, 14.9)	< 0.001	11.6 (8.3, 14.9)	< 0.001
Trauma diagnosis	0.6 (− 0.8, 2.0)	0.38		

Linear regression findings for factors associated with patient length of stay, with both univariate and multivariate findings

*CI* Confidence interval, *IC3* Integrated chronic care clinic, *SIRS* Systemic inflammatory response syndrome, *SBP* Systolic blood pressure, *HIV* Human immunodeficiency virus, *TB* Tuberculosis, *NCD* Non-communicable disease

In univariate analysis, factors associated with in-hospital mortality included age (odds ratio [OR] 1.03, *p* < 0.001), female sex (OR 0.5, *p* = 0.003), IC3 enrollment (OR 4.2, *p* < 0.001), SIRS on admission (OR 2.5, *p* < 0.001), hypoxia on admission (OR 4.4, *p* < 0.001), hypotension on admission (OR 7.7, *p* < 0.001), HIV diagnosis (OR 4.5, *p* < 0.001), TB diagnosis (OR 6.5, *p* < 0.001), malaria diagnosis (OR 0.3, *p* = 0.002), surgery during hospitalization (OR 0.1, *p* = 0.02), medical NCD diagnosis (OR 3.4, *p* < 0.001), number of medical NCD diagnoses (OR 2.0, *p* < 0.001), and trauma diagnosis (OR 0.3, *p* = 0.04) (Table 5). In multivariate analysis, factors that were positively associated with in-hospital mortality included age in years (OR 1.02, *p* < 0.001), hypoxia on admission (OR 3.6, *p* = 0.002) and hypotension on admission (OR 3.8, *p* = 0.001), HIV diagnosis (OR 4.5, *p* < 0.001), TB diagnosis (2.8, *p* = 0.02), and medical NCDs diagnosis (OR 1.0, *p* = 0.03). Female sex (OR 0.5, *p* = 0.01) was negatively associated with in-hospital mortality.

**Table 5:** Criteria associated with in-hospital mortality

Variable	Univariate analysis		Multivariate analysis	
	Odds ratio (95% CI)	*p* value	Odds ratio (95% CI)	*p* value
Age, years (per year)	1.03 (1.02, 1.04)	< 0.001	1.02 (1.01, 1.03)	< 0.001
Female sex (reference: male sex)	0.5 (0.3, 0.8)	0.003	0.5 (0.3, 0.8)	0.01
IC3-enrolled (reference: non-enrolled)	4.2 (2.4, 7.2)	< 0.001		
Rainy season admission (reference: dry season admission)	0.9 (0.6, 1.4)	0.77		
SIRS on admission (reference: no SIRS on admission)	2.5 (1.6, 3.9)	< 0.001	1.7 (1.0, 2.8)	0.06
Hypoxia (oxygen saturation < 90%) on admission (reference: no hypoxia on admission)	4.4 (2.2, 8.6)	< 0.001	3.6 (1.6, 8.0)	0.002
Hypotension (SBP < 90 mmHg) on admission (reference: no hypotension on admission)	7.7 (4.1, 14.5)	< 0.001	3.8 (1.8, 8.0)	0.001
HIV diagnosis (reference: HIV negative)	4.5 (2.9, 7.1)	< 0.001	2.8 (1.6, 5.0)	< 0.001
TB diagnosis (reference: TB negative)	6.5 (3.6, 11.8)	< 0.001	2.3 (1.1, 4.9)	0.02
Malaria diagnosis (reference: malaria negative)	0.3 (0.1, 0.6)	0.002		
Surgery during hospiatlization (reference: no surgery)	0.1 (0.01, 0.6)	0.02	0.2 (0.02, 1.2)	0.08
Medical NCD diagnosis (reference: no medical NCD diagnosis)	3.4 (2.2, 5.3)	< 0.001	1.9 (1.1, 3.3)	0.03
Number of medical NCD diagnoses (per unit)	2.0 (1.5, 2.6)	< 0.001		
Burns diagnosis (reference: no burns diagnosis)	1.8 (0.4, 7.7)	0.42		
Trauma diagnosis (reference: no trauma diagnosis)	0.3 (0.1, 0.9)	0.04		

Logistic regression findings for factors associated with patient in-hospital mortality, with both univariate and multivariate findings

*CI* Confidence interval, *IC3* Integrated chronic care clinic, *SIRS* Systemic inflammatory response syndrome, *SBP* Systolic blood pressure, *HIV* Human immunodeficiency virus, *TB* Tuberculosis, *NCD* Non-communicable disease

## Discussion

In this study, we found high rates of NCDs among inpatients in a district hospital in rural Malawi. We show significant numbers of NCDs outside the traditional set of cardiovascular disease, diabetes, respiratory diseases, and cancers. In particular, we found especially high rates of trauma, mental health, and neurological disease. We also found that length of stay was significantly associated with carrying a diagnosis of a medical NCD or being diagnosed with a burn. Additionally, the odds of in-hospital mortality in our cohort were 1.9-fold greater with a medical NCD diagnosis.

The mean age for all NCD visits was 37.6, and we found two distinct populations of patients. The first population were patients over 40 years of age, predominantly affected by chronic medical conditions featured in the ‘4 × 4’ set of NCDs put forward by the WHO. The second population were patients under age 40, who primarily suffered from trauma, burns, and mental health disorders, as well as other chronic conditions such as asthma and epilepsy. We also found a significant number of complex medical NCDs among the pediatric population, including sickle cell disease, rheumatic heart disease, and type 1 diabetes. The rates of burns and trauma in our cohort were higher than those found previously [9], but show the need for ongoing research into this area of need.

The association of carrying a medical NCD diagnosis with both longer length of stay and increased odds of in-hospital mortality supports prior studies that demonstrate the significant toll that NCDs take on patients and the health system in SSA [38, 39]. This is likely because NCD patients present with more advanced disease given inadequate outpatient follow-up, require increased hospital resources that may be lacking, and/or their comorbidities worsen the acute medical problem. To improve patient outcomes, we must take an approach that links high-quality acute care with improved longitudinal care in the outpatient setting. Given the high proportion of NCD patients on inpatient wards, there is a real opportunity for programs that screen inpatients for NCDs and then subsequently link them to high-quality outpatient care. These programs can capture NCDs beyond the traditional 4 × 4 agenda.

It was surprising that having more medical NCD diagnoses was associated with shorter length of stay. However, the effect was small, and this may have been skewed by the few patients with multiple medical NCDs in our cohort. It is also possible that carrying more diagnoses was related to better access to outpatient care, hence better access to diagnosis of chronic conditions. It was also surprising that trauma was not associated with length of stay or in-hospital mortality. This was likely because most traumas in our cohort were non-life-threatening orthopedic injuries. Further research into the long-term disability from trauma is critical to capture the full impact of trauma in Malawi. Burns were associated with long hospital stays in our cohort, with especially high burden among the pediatric population. This is in line with prior studies, and highlights the dire need for increased resources, training, and attention towards burn care in SSA [40].

The mortality in our cohort (4%) was lower than a similar cohort at Queens Hospital in Blantyre, Malawi, who had a mortality rate of 22.7% [21]. This is not necessarily surprising, as Queens is a referral hospital with an Intensive Care Unit and larger catchment area, likely resulting in a sicker patient population. However, the mortality difference may also be in part to patients presenting to Neno Hospital, a rural hospital closer to their home, earlier in their disease course, though we did not have enough data to evaluate this hypothesis. Additionally, there may be some positive effect from the IC3 clinic in Neno district, which provides longitudinal services free of charge to patients with HIV and chronic NCDs [26].

This study highlights that NCDs are an important part of the inpatient population in rural areas in Sub-Saharan Africa. While the rates of NCDs in our population were high, these rates are likely an underestimation. The diagnosis of many NCDs remains elusive in hospitals throughout Sub-Saharan Africa due to a lack of resources like consistent laboratory testing, spirometry, and reliable imaging in addition to a lack of training for staff. As an example, while the prevalence of RHD in Malawi is estimated at 1% [41], active screening diagnosed latent RHD in 3.4% of school-aged children [42].

A major part of the UHC effort is to match resources with burden of disease. Currently, public resources are often concentrated in urban areas, further exacerbating urban–rural health inequities [43]. The high rates of disease we found at Neno District Hospital highlight that rural hospitals must also be equipped to diagnose and care for a broad range of NCDs, especially those in young people. These diseases in young people are often not covered by the traditional 4 × 4 set. The significant numbers of neurological disease, mental health disorders, severe chronic conditions, as well as trauma and burns, show the importance of broadening our definitions of NCD to better address the true disease burden.

The major strength of this study is that it is the first to look at the burden of NCDs among inpatients at a rural hospital in Malawi. Additionally, we took a broad look at NCDs, not only focusing on diseases outside of the traditional 4 × 4 set as defined by WHO PEN, but also included the pediatric inpatient population.

A major weakness of this study was that it is a retrospective study with relatively small sample size, only evaluating 16 months of hospital admissions. There are also limitations to the ability to diagnosis all NCDs consistently in our hospital. Given a lack of resources and training, we had difficulty arriving at specific diagnoses for some pulmonary, cardiac, and other complex medical conditions. This is not unique to our study, and despite significant efforts in training and diagnostics, there remains progress to be made. This study highlights the importance of ongoing research into true burdens of non-communicable disease.

## Conclusions

In this study, we show high rates of NCDs in a district hospital in rural Malawi, with a significant proportion outside of the typical 4 × 4 set as put forward by WHO PEN. We also found high rates of NCDs among young patients (under age 40) with a significant proportion of trauma and burns. Additionally, length of stay was associated with carrying a diagnosis of a medical NCD or burns and patients with medical NCDs had higher odds of in-hospital mortality. This study highlights the need for increased resources and training around NCD care to better support patients and robust programs to link inpatients to longitudinal outpatient systems of care. Future research is needed to look at disease severity and the impacts of NCD clinical programs on patient outcomes.

## Supplementary Information

**Additional file 1. Figure S1.** Age distribution of patients with or without an NCD diagnosis. Box plot of age distribution of patients who did or did not carry an NCD diagnosis. Boxes show the 25th and 75th percentiles, and whiskers convey the upper and lower adjacent values, with dots representing outliers. *NCD* Non-communicable disease. **Figure S2.** Age distribution of patients by number of unique NCD diagnoses. Box plot representing the age distribution for the number of total NCD diagnoses a patient carried. Boxes show the 25th and 75th percentiles, and whiskers convey the upper and lower adjacent values, with dots representing outliers. *NCD* Non-communicable disease.

## Data Availability

The datasets used and/or analyzed during the current study are available from the corresponding author on reasonable request.

## Abbreviations

APZU: Abwenzi Pa Za Umoyo
CHF: Congestive heart failure
CKD: Chronic kidney disease
COPD: Chronic obstructive pulmonary disease
DALY: Disability-adjusted life year
HIV: Human immunodeficiency virus
IC3: Integrated Chronic Care Clinic
NCD: Non-communicable disease
NCDI: Non-communicable diseases and injuries
PIH: Partners in health
RHD: Rheumatic heart disease
SBP: Systolic blood pressure
SD: Standard deviation
SIRS: Systemic inflammatory response syndrome
SSA: Sub-Saharan Africa
TB: Tuberculosis
UHC: Universal Health Coverage
WHO: World Health Organization
WHO PEN: WHO Package of Essential Noncommunicable Disease Interventions
