# Supplementary material for: Non-communicable disease burden among inpatients at a rural district hospital in Malawi
Source: Glob Health Res Policy. 2023 Feb 22;8:4. doi: 10.1186/s41256-023-00289-z (PMC9945353; doi:10.1186/s41256-023-00289-z)
Supplement: Supplementary file 1 — Additional file 1. Figure S1. Age distribution of patients with or without an NCD diagnosis. Box plot of age distribution of patients who did or did not carry an NCD diagnosis. Boxes show the 25th and 75th percentiles, and whiskers convey the upper and lower adjacent values, with dots representing outliers. NCD Non-communicable disease. Figure S2. Age distribution of patients by number of unique NCD diagnoses. Box plot representing the age distribution for the number of total NCD diagnoses a patient carried. Boxes show the 25th and 75th percentiles, and whiskers convey the upper and lower adjacent values, with dots representing outliers. NCD Non-communicable disease. [file 41256_2023_289_MOESM1_ESM.docx]

**Supplementary Figure 1: Age distribution of patients with or without an NCD diagnosis.** Box plot of age distribution of patients who did or did not carry an NCD diagnosis. Boxes show the 25^th^ and 75^th^ percentiles, and whiskers convey the upper and lower adjacent values, with dots representing outliers. NCD: non-communicable disease.

**
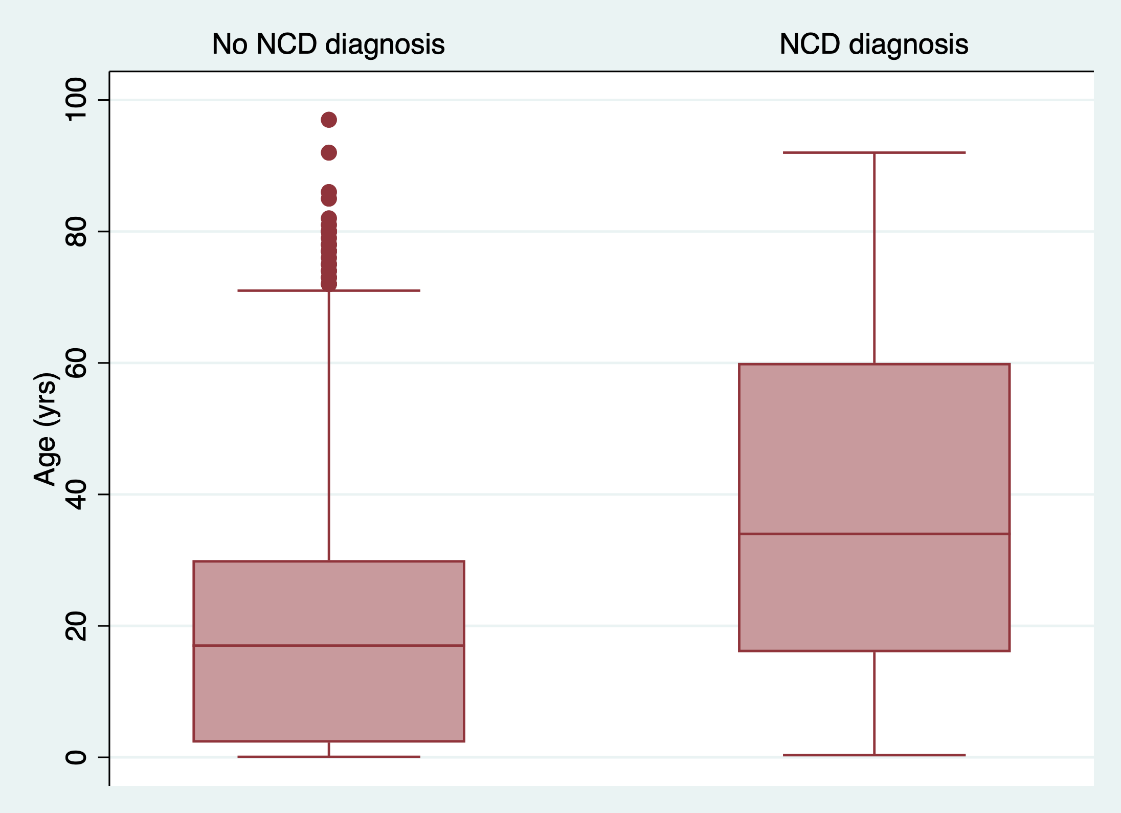
**

**Supplementary Figure 2: Age distribution of patients by number of unique NCD diagnoses.** Box plot representing the age distribution for the number of total NCD diagnoses a patient carried. Boxes show the 25^th^ and 75^th^ percentiles, and whiskers convey the upper and lower adjacent values, with dots representing outliers. NCD: non-communicable disease.


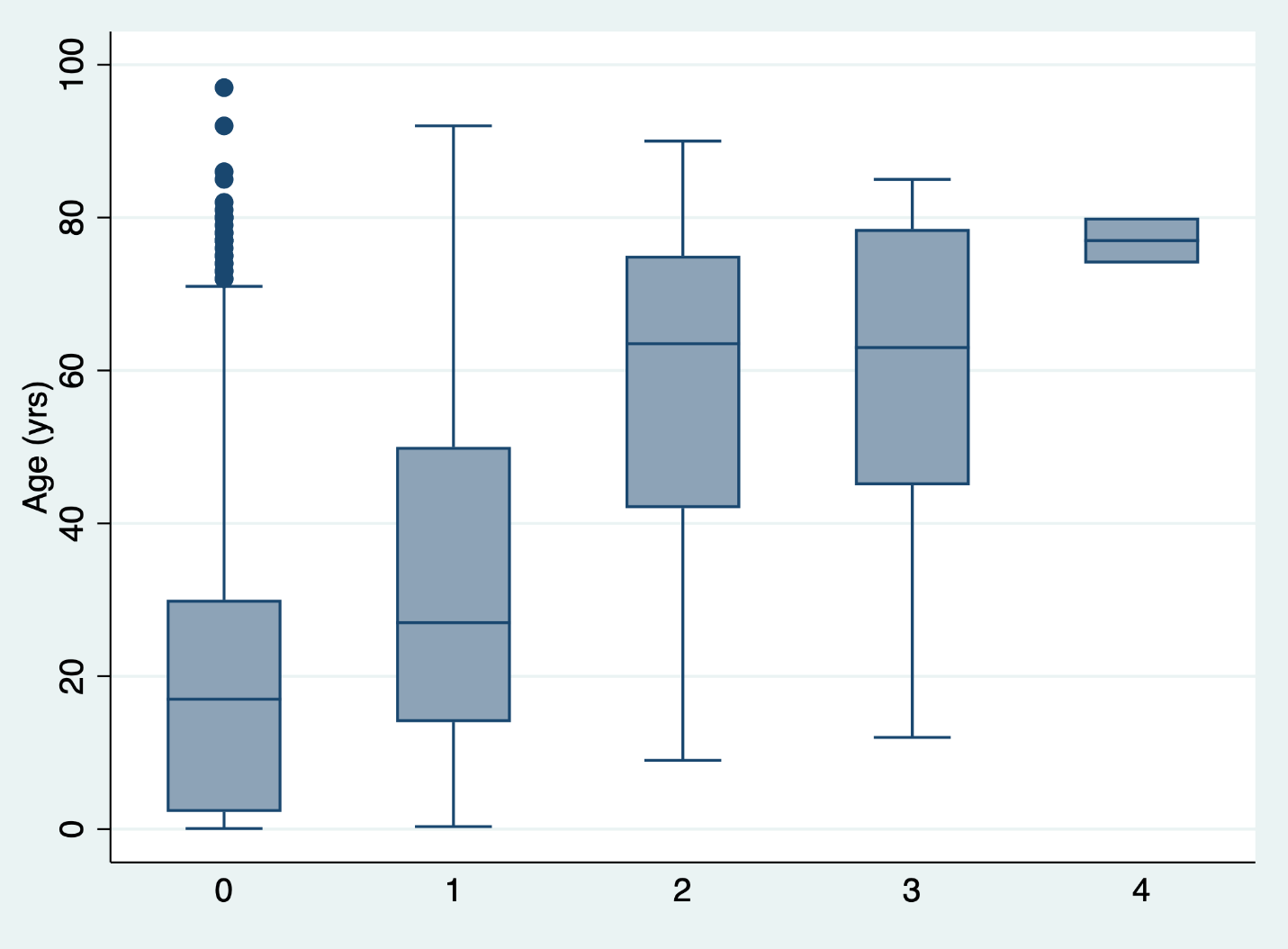


Number of unique NCD diagnoses
